# Structural enrichment for laboratory mice: exploring the effects of novelty and complexity

**DOI:** 10.3389/fvets.2023.1207332

**Published:** 2023-09-29

**Authors:** Lena Bohn, Louisa Bierbaum, Niklas Kästner, Vanessa Tabea von Kortzfleisch, Sylvia Kaiser, Norbert Sachser, S. Helene Richter

**Affiliations:** ^1^Department of Behavioural Biology, Institute of Neuro- and Behavioural Biology, University of Münster, Münster, Germany; ^2^Münster Graduate School of Evolution, University of Münster, Münster, Germany

**Keywords:** refinement, enrichment, animal welfare, housing condition, complexity, novelty

## Abstract

Providing structural enrichment is a widespread refinement method for laboratory rodents and other animals in captivity. So far, animal welfare research has mostly focused on the effect of increased complexity either by accumulating or combining different enrichment items. However, increasing complexity is not the only possibility to refine housing conditions. Another refinement option is to increase novelty by regularly exchanging known enrichment items with new ones. In the present study, we used pair-housed non-breeding female C57BL/6J and DBA/2N mice to investigate the effect of novelty when applying structural enrichment. We used a double cage system, in which one cage served as home cage and the other as extra cage. While the home cage was furnished in the same way for all mice, in the extra cage we either provided only space with no additional enrichment items (space), a fixed set of enrichment items (complexity), or a changing set of enrichment items (novelty). Over 5  weeks, we assessed spontaneous behaviors, body weight, and extra cage usage as indicators of welfare and preference. Our main results showed that mice with access to structurally enriched extra cages (complexity and novelty) spent more time in their extra cages and complexity mice had lower latencies to enter their extra cages than mice with access to the extra cages without any structural enrichment (space). This indicates that the mice preferred the structurally enriched extra cages over the structurally non-enriched space cages. We found only one statistically significant difference between the novelty and complexity condition: during week 3, novelty mice spent more time in their extra cages than complexity mice. Although we did not detect any other significant differences between the novelty and complexity condition in the present study, more research is required to further explore the potential benefits of novelty beyond complexity.

## Introduction

Environmental enrichment is a major tool to refine housing conditions for captive animals. Since the benefits of environmental enrichment have been first described (1), the effects of different types of sensory, social, and structural enrichment have been investigated, covering a variety of different species living in laboratories, in zoos, and on farms [e.g., (2–9)]. Still, the main body of research on environmental enrichment has been conducted using laboratory rodents (10, 11).

While the results between the studies sometimes vary depending on the strain, sex, age, and differences in exposure to environmental enrichment (12–15), they support the general notion that environmental enrichment can increase the animals’ welfare. The wide range of reported beneficial effects through environmental enrichment include (but are not limited to): increased neurogenesis, learning and memory performance (16–19); promotion of species-specific behaviors and preventing the occurrence of behavioral disturbances like stereotypies (10, 20–24); facilitation of development of individual variation (25–27); as well as the reduction and mediation of anxiety, depression, and stress (17, 28–31). Together with the already mentioned factors, preference can likewise be used to assess welfare (32–34), and indeed, rodents show a preference for increased complexity and are even willing to work for access to additional enrichment (35).

Yet, given that laboratory rodents adapt very quickly to new conditions and environments, an initially beneficial impact of increased complexity might cease over time. One simple way of providing not only complexity but also novelty is to regularly exchange enrichment items, for example as part of the cage-changing routine. Indeed, researchers conducting enrichment studies have used novelty as part of their enrichment. However, it is impossible to disentangle whether reported welfare effects or preferences were due to the increased complexity or the novelty. That is because novelty was not systematically applied as a distinct enrichment strategy. Rather, novelty was used as part of the complex housing condition to further increase the contrast between the enriched and unenriched environments [see e.g., (11, 25, 36–38)].

So far, only a few studies have focused specifically on the effect of novelty on the welfare of laboratory rodents. For example, Abou-Ismail and Mendl (39) housed rats under two conditions, labeled complexity and novelty. In the novelty condition, the rats were provided five copies of the very same enrichment item, e.g., five ladders, which were exchanged weekly for five copies of another enrichment item, e.g., five shelters. In contrast, the rats from the complexity condition were offered five different items at a time which were not exchanged over the course of the 5 weeks. They found the complexity condition preferable over the novelty condition. Notably, their novelty condition did not always offer shelter or nesting material. Because nesting material has been identified as a major enrichment item for laboratory rodents (10, 40), their novelty housing condition without nesting material can arguably be considered impoverished compared to their complexity condition which always offered nesting material. In another experiment performed with mice, Gross et al. (41) did not find beneficial effects of novelty either. In their study, the shelters remained inside the cages for the duration of the experiment in the complexity housing condition, but in the novelty housing condition the shelters were replaced with a different kind of shelter every week (41). However, it has previously been argued that novel objects, especially when introduced into the home cage, might be perceived as a threat (42). Hence, to avoid potential adverse effects of novelty, it would be more cautious to avoid applying novel objects to the home cage but find ways to provide novelty outside the home cage (e.g., in an accessible extra cage or a play chamber).

With the present study, we aimed to further explore the potential benefits of novelty when providing structural enrichment. We compared the effects of three different enrichment conditions on mice, the most commonly used model species (43, 44). In contrast to the aforementioned studies, we used a double cage system (45), in which one cage served as home cage and the second one was used as extra cage. This system allowed us to provide the mice constant access to nesting material and shelter in their home cage, and additionally, to offer novelty in a voluntarily accessible extra cage outside the familiar home cage. To systematically compare the effects of novelty and complexity on the mice, the extra cages either provided a weekly changing set of enrichment items (novelty), a fixed set of enrichment items (complexity), or no additional enrichment items (space). To gain insights regarding the effects of enrichment condition on the mice’s welfare and their preferences, we monitored the mice’s spontaneous behavior, body weight, and extra cage usage. More specifically, if for example, novelty were superior to complexity, we would expect to see more behaviors indicative of good welfare, e.g., more play, and fewer signs of poor welfare, e.g., fewer stereotypies, in the novelty mice (32, 46, 47), and if for example, novelty were more attractive to the mice than mere complexity, we would expect that novelty mice enter the extra cages faster when access is provided and spend more time in the extra cage, as well as interacting more with the enrichment therein.

## Methods

### Animals and housing condition

We purchased 18 female C57BL/6J and 18 female DBA/2N mice from a professional breeder (Charles River Laboratories, Germany GmbH, Sulzfeld, Germany) at the age of post-natal day (PND) 28.

After arrival and prior to the present study, the mice participated in another experiment (45). In brief, the mice’s previous experience included different housing conditions as well as behavioral tests. Upon arrival, the mice were pair-housed in either same-strain or mixed-strain pairs in Makrolon type III cages (39 × 23 × 15 cm^3^). The cage floor was covered with wood shavings (Tierwohl, Wilhelm Reckhorn GmbH & Co. KG, Warendorf, Germany). Food (Altromin 1,324, Altromin Spezialfutter GmbH & Co. KG, Lage, Germany) and water were provided *ad libitum*. The cages were furnished with a paper towel, a wooden gnawing stick, a red transparent plastic house (Mouse House™, Tecniplast Deutschland GmbH, Hohenpeißenberg, Germany), and a red transparent plastic tunnel (Mouse Tunnel Red, Plexx B.V., Elst, Netherlands), which was attached to the cage lid via wire hangers (Stainless Steel wire Hanger for Mouse Tunnel, Plexx B.V., Elst, Netherlands). On PNDs 76–92, the mice were tested on the elevated plus maze, in the dark–light test, the open field test, the free exploration test, and in a labyrinth. On PND 97, always two pairs were merged to form quartets (two C57BL/6J mice and two DBA/2N mice) and were transferred into a double cage system. The double cage system consisted of two Makrolon type III cages, which were furnished as described above and were connected via a transparent tunnel (length: 8.4 cm, diameter: 3.9 cm). The tunnel was closable using a gray PVC platelet (height: 4.9 cm, width: 3.4 cm). On PND 157, the groups were divided into mixed-strain pairs and transferred to single Makrolon Type III cages.

At the beginning of the present study on PND 167, the mixed-strain pairs were transferred from single Makrolon type III cages into double cage systems. One of the two cages served as home cage and was furnished as described above minus the red mouse house, the second cage served as extra cage and will be described in detail below. Enrichment and bedding were changed weekly (paper tissue, bedding) or biweekly (tunnel, wooden gnawing stick). We decided to pair-house one C57BL/6J and one DBA/2N mouse because results from previous studies suggested that the effects of environmental enrichment can be strain-specific (44, 48, 49) and the use of more than one strain allows for a greater generalization of the results (12, 50). We decided on these two strains in particular because they are widely used in research (44, 48), their different coats allow for individual recognition during the behavioral observations, and previous studies showed that these two strains can be housed together harmoniously (51). All experiments and observations were carried out in the dark phase of the inversed 12 h/12 h dark–light cycle. Room temperature and humidity were kept around 22°C and 50%, respectively. The 18 double cage systems were distributed over three racks in a balanced way to account for systematic differences within the housing rooms, especially regarding light conditions and human traffic.

### Experimental design

**Figure 1:**
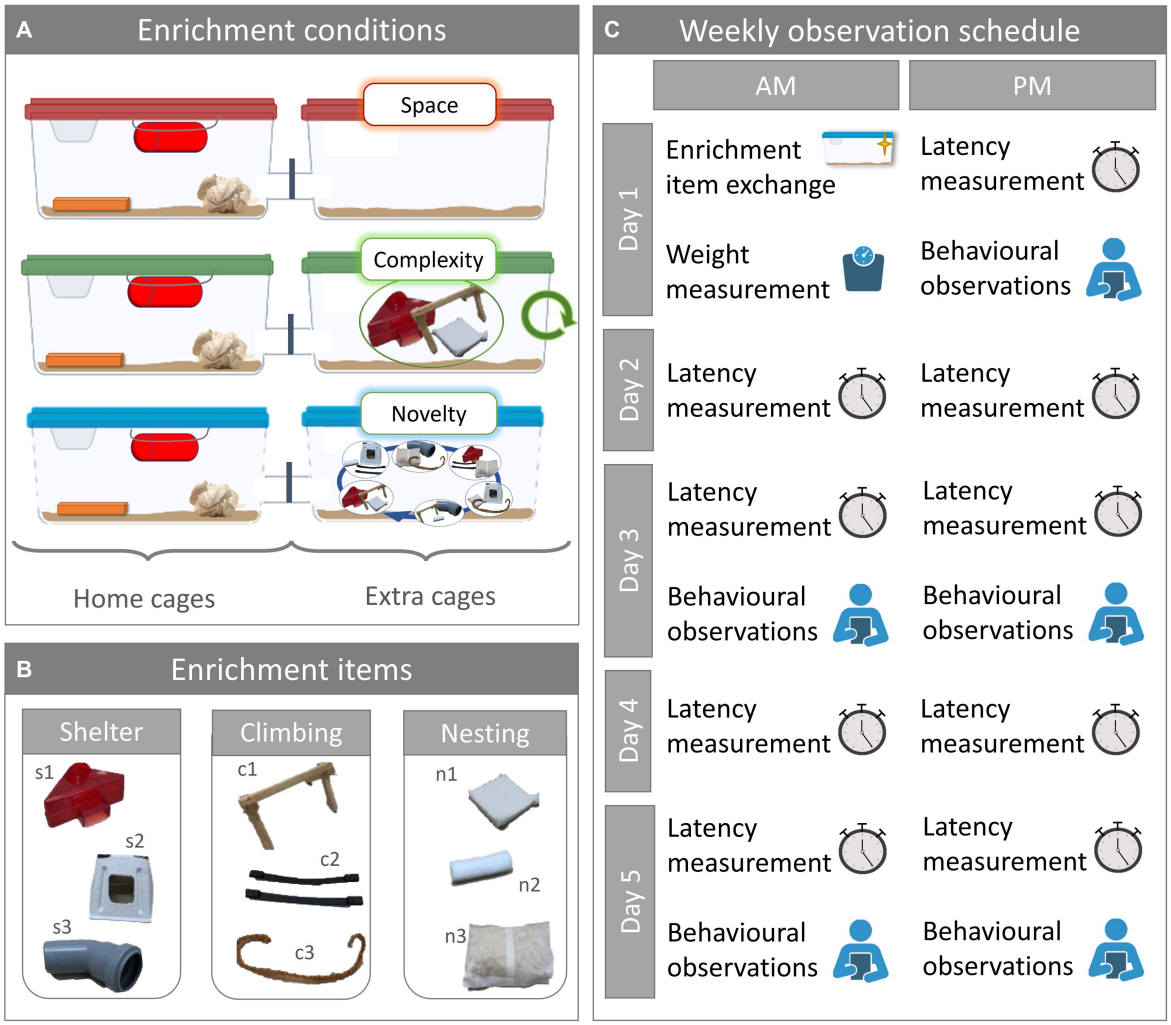
Experimental design. **(A)** Enrichment conditions. Mice were housed in mixed strain pairs in double cage systems and were assigned to one of three enrichment conditions designated as space, complexity, or novelty. One of the cages served as home cage and was furnished the same way for all enrichment conditions. The second cage served as extra cage and was furnished depending on the enrichment condition: extra cage space did not contain any additional enrichment; extra cage complexity offered a fixed set of additional three enrichment items; extra cage novelty offered a changing set of three additional enrichment items. Total sample size was *N* = 36, with *n* = 6 per group (N_C57BL/6J, space_ = N_C57BL/6J, complexity_ = N_C57BL/6J, novelty_ = N_DBA/2N, space_ = N_DBA/2N, complexity_ = N_DBA/2N, novelty_ = 6). **(B)** Enrichment items. The enrichment items used in the extra cages can be grouped into three categories: shelter, climbing, and nesting. For shelter, we used red transparent plastic houses (s1), cardboard houses (s2), and gray opaque PVC tubes (s3). For climbing we used wooden scaffolds (c1), mouse swings (c2), and hemp rope (c3). For nesting we used nestlets (n1), cocoons (n2), and nest packs (n3). **(C)** Weekly observation schedule. Once a week on Day 1, enrichment items were exchanged, and the mice were weighed. In the afternoon of Day 1 and twice on every other weekday, latency to enter the extra cage was measured. On five occasions, the latency measurement was followed by behavioral observation sessions.

To explore the effect of enrichment novelty on the mice, we assigned the 18 cages to either of three enrichment conditions: space, complexity, or novelty (Figure 1A). In the space enrichment condition, the extra cage was empty apart from the bedding material. In the complexity and novelty enrichment conditions, the extra cage was furnished with three different enrichment items. While for the complexity enrichment condition the set of enrichment items remained the same and the items were merely replaced by clean ones, mice in the novelty enrichment condition were presented with a different set of items every week.

We used enrichment items of three categories: shelter, climbing, and nesting. For each category, we used three different enrichment items (Figure 1B). In category shelter, we used the following items: red transparent plastic houses (s1; 11.1 × 11.1 × 5.5 cm^3^; Tecniplast Deutschland GmbH, Hohenpeißenberg, Germany), cardboard houses (s2; 13 × 9 × 6 cm^3^; ZOONLAB GmbH, Castrop-Rauxel, Germany), and opaque gray PVC tunnels (s3; 3.5 × 10 cm; Bauhaus AG, Belp, Schweiz). In category climbing, we used: wooden scaffolds (c1; 11 × 14 × 22 cm), nylon mouse swings (c2; PLEXX B.V., Elst, Netherlands), and hemp rope (c3). In category nesting, we used: nestlets (n1; 5 × 5 cm^2^; ZOONLAB GmbH, Castrop-Rauxel, Germany), cocoons (n2; 3.6 × 1.2 cm; ZOONLAB GmbH, Castrop-Rauxel, Germany), and nest packs (n3; 100 g; ZOONLAB GmbH, Castrop-Rauxel, Germany). The extra cage enrichment was replaced during the regular cage-changing routine on the first day of the week (day 1) during the morning (Figure 1C). For the present study, we used 6 different combinations of enrichment items. Each of the six cages assigned to the complexity enrichment condition had one of the six combinations in their extra cage throughout the experiment. The six extra cages of the novelty enrichment condition each started with a different one of the six possible combinations. In the following weeks, the combination of enrichment items in the novelty extra cages was changed.

Over the course of 5 weeks, we assessed body weight, extra cage usage, and spontaneous behaviors (Figure 1C). We measured the mice’s weight once a week during the cage-changing routine on Day 1, using a digital scale (CM 150-1 N, Kern, Ballingen, Germany; weighing capacity: 150 g, resolution: 0.1 g). To assess extra cage usage, we measured the mice’s latency to enter the extra cage, the relative time they spent in the extra cage, as well as enrichment item interaction frequency in the extra cage (only for the complexity and novelty enrichment condition). We measured the latency nine times per week: once in the afternoon of day 1 and twice per day on the following weekdays. We measured the latencies for both mice at the same time, immediately after we opened the connection tunnel, using stopwatches, for a maximal latency of 180 s. Five times per week, we conducted behavioral observations after all the latencies had been taken: Once on day 1 and twice on days 3 and 5. During each behavioral observation, we recorded the spontaneous behaviors (Table 1A) and enrichment item interactions in the extra cage by counting the number of events (Table 1B), and by measuring the time the mice spent in the extra cage using stopwatches. The mice of one cage were observed separately but immediately after one another before moving on to the next cage, alternating with the starting mouse between observation sessions. Each observation lasted for 60s, except on the very first day when observations lasted for 90s (the data have been corrected accordingly in the analysis). To have a more balanced observation, we split the 60s (or 90s) observations in two intervals: After the first 30s (or 45s) for each of the two mice in one cage, we moved on to the next cage until all mice of all cages were observed once before we returned to the first cage to observe the mice for the second interval. The observations started either with cage 1 and finished with cage 18 going forward through the rows, started with cage 18 and finished with cage 1 going backwards, or started in the middle and continued forwards.

**Table 1:** Ethogram.

(A) Spontaneous behaviors		
Lid climbing		The mouse grabs the grid of the cage lid with at least two paws without touching the ground or the mouse house and moves along it.
Digging		The mouse shoves bedding material under its body using its forepaws in alternating movement. The hind paws may push the bedding material behind the mouse. Alternatively, it pushes the bedding material in front of its body showing a forward locomotion.
Bar-mouthing		The mouse holds a bar of the cage lid in its mouth for at least 3 s without an interruption longer than 1 s. Biting movements may be seen.
Inactivity		The mouse does not move for the whole length of the observed interval except for breathing or tiny ear or whisker movements.
Play (14)	Hopping	The mouse suddenly (without identifiable reason) jumps vertically. The behavior is often accompanied by head shaking.
	Jumping	The mouse suddenly (without identifiable reason) jumps horizontally, not shorter than the length of a mouse.
Agonistic behavior (52)	Chasing	The mouse approaches another mouse which then runs away while being followed by the focal mouse.
	Mounting	The mouse lays its upper body on the back of another mouse. The front paws grab the sides of the body of the recipient mouse. The mouse may show pelvic thrusts.
	Fighting	The mouse bites, kicks, and wrestles another mouse in fast movements. The recipient mouse may produce squeaking noises.
Stereotypic behavior	Route-tracing	The mouse moves along an identical path on the cage lid or bottom for at least three times in a row. Circular paths are excluded.
	Circling	The mouse moves along an identical circular path for at least three times in a row.
(B) Extra cage enrichment item interaction		
Shelter	In	The mouse moves into the shelter or stays within. The whole body is under the roof of the shelter, only the tail may be outside.
	On	The mouse is sitting, standing, or moving along the top of the shelter. At least two paws need to touch it without touching the bottom or different objects.
	Gnawing	The mouse touches the shelter with its mouth for at least 2 s. A slight movement of the head is visible.
Climbing item	On	The mouse is sitting, standing, or moving along the top of the climbing item. At least two paws touch it without touching the bottom or different objects.
	Gnawing	The mouse touches the shelter with its mouth for at least 2 s. A slight movement of the head is visible.
Nesting material	Carrying	The mouse holds the nesting material in its mouth and is moving around the cage.
	Manipulating	The mouse touches the nesting material with its mouth and pushes or tears it apart without showing locomotion.

On five live observations per week, we recorded the mice’s spontaneous behavior (A) and enrichment interaction in the extra cage (B) by counting the events.

For the behavioral observations we used continuous recording except for inactivity, for which we used one–zero sampling (53). After the second observation interval, the observations for a given cage were over, the mice were gently guided back into their home cage and the connection tunnels were closed again. This design led to slightly different extra cage access times, as they were depending on the latencies of the other mice. The mice had access to the extra cages twice a day (except for Day 1 of the week, where they had access once in the afternoon) for roughly 90 min each time, starting with the opening of the tunnels before the latency measurement until the closing of the tunnel at the end of the second observation interval.

All observations were performed by the same observer. Due to the nature and design of the experiment, it was not possible to blind the observer to the enrichment condition.

### Statistical analysis

To statistically analyze our data, we fitted linear mixed-effect models (LMMs) or used non-parametric tests, depending on our outcome measures.

We fitted two LMMs with three fixed factors to analyze the effect of enrichment condition (factor with three levels: space, complexity, and novelty), strain (factor with two levels: C57BL/6J and DNA/2N), and week (numeric factor) on latency to enter the extra cage and on relative body weight. Relative body weight captures individual weight changes better than absolute body weights; therefore, we set the first measurement (week 1) as the baseline value and divided the following measurements (week 2 onwards) by the baseline value. In addition to the main effects, the models included two interactions, namely enrichment condition*strain and enrichment condition*week, as well as animal ID as random factor. As reference levels for our fixed factors enrichment condition and strain, we used space enrichment condition and C57BL/6J, respectively. In case of significant results from the ANOVA of the fitted model, we conducted Tukey-adjusted pairwise comparisons as *post hoc* analysis.

Our other outcome measures, namely relative time spent in the extra cage, extra cage enrichment item interaction frequency, inactivity, and spontaneous behaviors could not be fitted by LMMs without violating model assumptions, even after transformation of the raw data. Relative time spent in the extra cage was calculated by dividing the time the mice spent in the extra cage by the time the mice were observed. This was done to account for differences in observation durations (90s or 60s). To analyze the effect of enrichment condition on our outcome measures, we used Kruskal-Wallis rank sum tests. However, for the extra cage enrichment item interaction frequency, we used Mann–Whitney-U tests because we only had the novelty and complexity enrichment condition to compare (the space enrichment condition did not offer any enrichment items in the extra cage). We tested for an overall enrichment condition effect by comparing mouse means from both strains across all 5 weeks, as well as testing for each week separately.

Even though this was not in the focus of the present study, we also tested for strain and week effects, to better match the non-parametric analysis with the LMMs described above. To analyze the effect of strain on our outcome measures, we used Mann–Whitney-U tests. To analyze the effect of week on our outcome measures, we used Friedman rank sum tests.

To account for multiple hypothesis testing in analysis, we used sequential Bonferroni-Holm correction (54) to adjust the *p*-values from the Kruskal-Wallis, Mann–Whitney-U, and Friedmann tests as well as the *p*-values from the *post hoc* pair-wise comparisons.

Due to the two different approaches in the analysis (LMMs and non-parametric tests), there are differences in the way the results are reported and displayed. In our analysis, week is a numeric factor in the LMMs but a five-level factor in our non-parametric Friedman rank sum tests. Hence, we needed to conduct *post hoc* pairwise comparisons for a significant effect of week following the Friedman ranks sum tests, while this was not necessary for the LMMs. Likewise, in our plots, we display the values which we used for the analysis. As we used every single value for the analysis of latency to enter the extra cage and relative body weight, we also used all the values when creating the plot. For the non-parametric tests, on the other hand, we used individual means, hence the plots display individual means rather than every value for each individual.

Across all three enrichment conditions, some mice did not enter the extra cage during the observation time. This was accounted for in the analysis as follows: for latency to enter the extra cage, observations in which the mice did not enter the extra cage were excluded from the analysis; for time spent in the extra cage, observations in which the mice did not enter the extra cages scored 0; for enrichment item interaction frequency in the extra cage, observations in which mice did not enter the extra cage were excluded from the analysis, as we divided the number of extra cage enrichment item interactions by the time the mice spent in the extra cage (and division by 0 is an invalid operation).

All statistical analyses were performed in R version 4.1.2 (55). To fit the LMMs, we used the lme4 (56) and lmerTest packages (57). For the *post hoc* analysis, we used the emmeans package (58) and the dunn.test package (59). To test model assumptions like normal distribution of model residuals, we used the packages nortest (60) and performance (61). For the Tukey transformation of the latency data, we used the rcompanion package (62). We used the packages ggplot2 (63) and ggpubr (64) to create the figures. We considered a value of *p* < 0.05 as an indicator of statistically significant differences. For easier reading, we simply referred to “differences” instead of “statistically significant differences” in the results section.

In addition to our statistical analysis in R, we also conducted a sensitivity analysis using the software G*Power (65), with an α error probability = 0.05 and β error probability = 0.2, to determine the detectable effect sizes for the LMMs. Our sensitivity analysis revealed that the detectable effect size for enrichment condition was Cohen’s *f* = 0.542, which is above what is considered to be the threshold for a large effect size (for more details, please see Supplementary Table S8) (66, 67).

## Results

To test whether enrichment condition influenced the mice’s spontaneous behavior, body weight, and usage of the extra cage, we conducted behavioral observations and monitored the mice’s weight over the course of 5  weeks. We indeed found that enrichment condition influenced some of our outcome measures, namely relative time the mice spent in the extra cage, latency to enter the extra cage, and relative body weight.

**Figure 2:**
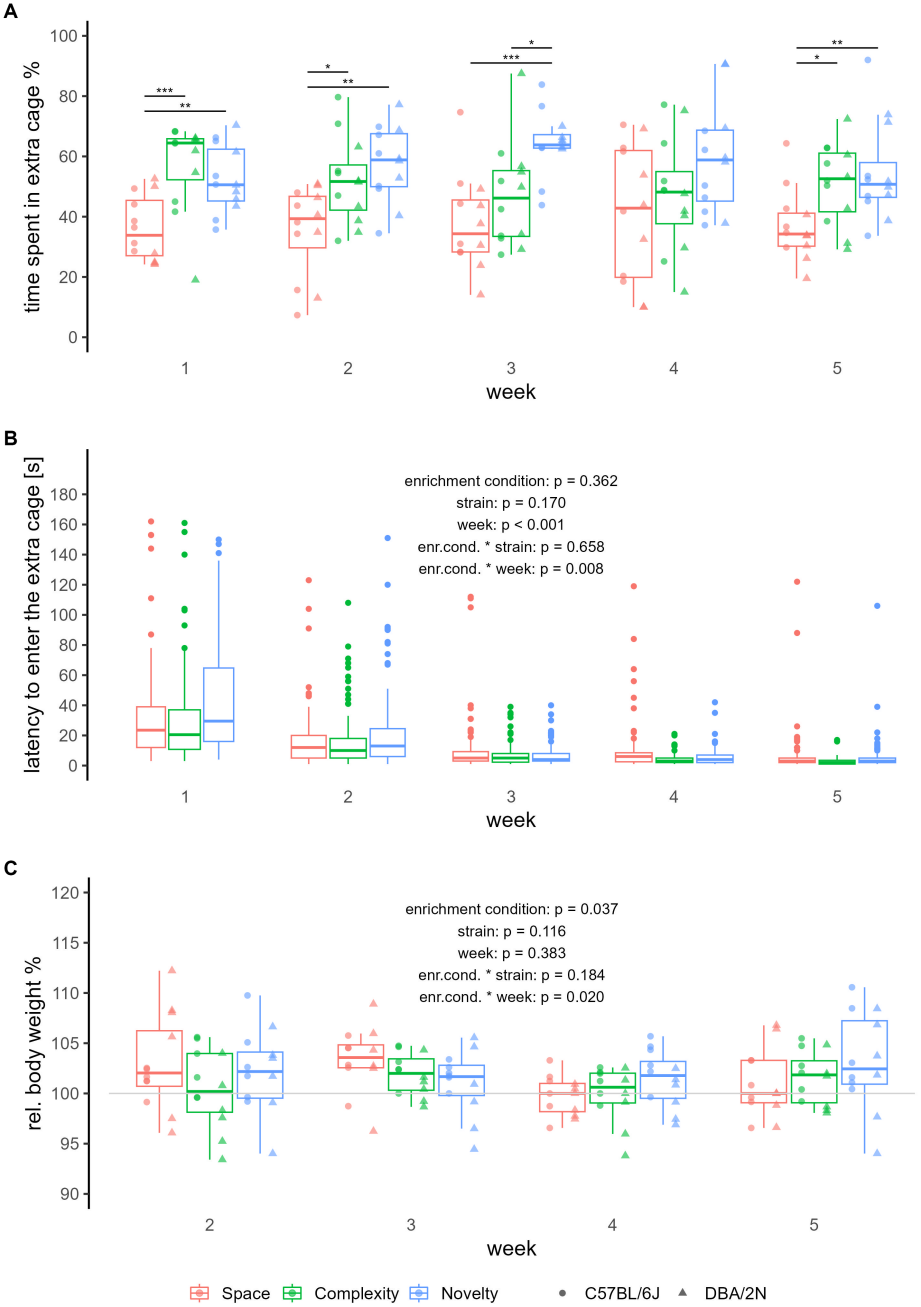
Relative time spent in the extra cage, latency to enter the extra cage, and relative body weight. The boxplots show the median, upper and lower quartile of the respective group. The colours refer to enrichment condition (red = space, green = complexity, blue = novelty), the dot shapes in **(A,C)** refer to strains (round = C57BL/6J, triangular = DBA/2N). We used N_C57BL/6J, space_ = N_C57BL/6J, complexity_ = N_C57BL/6J, novelty_ = N_DBA/2N, space_ = N_DBA/2N, complexity_ = N_DBA/2N, novelty_ = 6 individuals per group, all non-breeding females. Where no *p*-values are given, significance levels are indicated as follows: *p_adjusted_ ≤ 0.05, **p_adjusted_ ≤ 0.01, ***p_adjusted_ ≤ 0.001. **(A)** Each dot represents the average time spent in the extra cage of one individual each week, with 10 measurements per individual and week. Indicated are the statistically significant differences between the groups after the Kruskal-Wallis *post hoc* pairwise comparison using Dunn tests with Bonferroni-Holm adjustment. For the analysis of latency to enter the extra cage **(B)** and relative body weight **(C)**, we fitted linear mixed-effect models. Hence, **(B,C)** represent all measurements per individual, **(B)** with a max of 60 measurements per enrichment condition and week (measurements of >180 s were omitted), and **(C)** with a total of 12 measurements per enrichment condition and week. The weight measure of Week 1 was taken as the baseline value (horizontal line).

Regarding relative time spent in the extra cage, we see that across all 5  weeks, mice from the complexity and novelty enrichment condition spent more time in their extra cages than mice from the space enrichment condition (Kruskal-Wallis test for Weeks 1–5: χ^2^ = 16.294, *p* < 0.001; *post hoc* pairwise comparison for Weeks 1–5, complexity-space: Z = 2.519, *p* _adjusted_ = 0.012, novelty-space: Z = 3.991, *p* _adjusted_ < 0.001; Supplementary Table S1 and Figure 2A). This effect of enrichment condition on relative time spent in the extra cage, with mice spending more time in the structurally enriched extra cages compared to the structurally non-enriched extra cages from the space enrichment condition, can also be seen for all individually analyzed weeks, except during week 4 (for details please see Supplementary Table S1 and Figure 2A). The only time we detected a difference between the novelty and complexity enrichment condition was during week 3 (Kruskal-Wallis test for Week 3: χ^2^ = 14.114, *p* _adjusted_ = 0.005; *post hoc* pairwise comparison complexity-novelty: *Z* = −2.558, *p* _adjusted_ = 0.011, Supplementary Table S1 and Figure 2A), showing that mice from the novelty enrichment condition spent more time in the extra cage than mice from the complexity enrichment condition.

Regarding latency to enter the extra cage, we found a statistically significant interaction between enrichment condition and week (LMM: *F*_2,1429.553_ = 4.828, *p* = 0.008, Supplementary Table S2 and Figure 2B). *Post hoc* analysis indicated that over the weeks, the latency to enter the extra cage decreased significantly faster for mice from the complexity enrichment condition compared to mice from the space enrichment condition (*post hoc* pairwise comparison, space-complexity: *b* = −0.012 ± 0.004, *t*_1429.334_ = 2.956, *p* _adjusted_ = 0.009, space-novelty: *b* = 0.010 ± 0.004, *t*_1429.702_ = −2.345, *p* _adjusted_ = 0.050, Supplementary Table S3 and Figure 2B).

Regarding relative body weight, we also found a statistically significant interaction between enrichment condition and week (LMM: *F*_2,105_ = 4.04, *p* = 0.020, Supplementary Table S2 and Figure 2C). Here, *post hoc* analysis revealed differences in the slopes for the week effect on mice from the space enrichment condition and mice from the novelty enrichment condition, indicating a decrease in relative body weight over the weeks in space mice compared to mice from the novelty enrichment condition (*post hoc* pairwise comparison: *b* = −1.290 ± 0.491, *t*_105_ = −2.625, *p* _adjusted_ = 0.027, Supplementary Table S3 and Figure 2C). We also found a statistically significant effect of enrichment condition on relative body weight (LMM: *F*_2,134.1_ = 3.37, *p* = 0.037, Supplementary Table S2 and Figure 2C), but *post hoc* analysis revealed no statistically significant effect after correcting for multiple comparisons (for details please see Supplementary Table S3 and Figure 2C). For all other outcome measures, we did not find statistically significant effects of enrichment condition on our outcome measures (for more details please see Supplementary Table S1 and Supplementary Figure S1).

Albeit not the focus of this study, we also analyzed the effect of strain and week on our outcome measures. We found that week influenced latency to enter the extra cage. Apart from the interactive effect of week with enrichment condition described above, week had a statistically significant main effect on latency to enter the home cage (LMM: *F*_1,1429.553_ = 1004.212, *p* < 0.0001, Supplementary Table S2 and Figure 2B), showing that the latency to enter the extra cage decreased over the weeks. For all other outcome measures, we did not find statistically significant effects of week or strain after *post hoc* pairwise comparisons and correcting for multiple comparisons (for more details please see Supplementary Tables S4–S6 and Supplementary Figure S1).

Because of their rare occurrence, some spontaneous behaviors were only descriptively analyzed, namely agonistic behavior, stereotypic behaviors, and bar mouthing. Upon visual inspection of the data, we did not detect an effect of enrichment condition (for more details, please see Supplementary Table S7 and Supplementary Figure S2).

## Discussion

The present study aimed to explore whether novelty of environmental enrichment may offer beneficial effects beyond that of structural complexity alone. To this end, we allowed mice of two strains access to an extra cage offering either novelty, complexity, or space. Over the course of 5 weeks, we recorded spontaneous behaviors and body weight, as well as extra cage usage as indicators of welfare. With one exception during week 3, in which mice from the novelty condition spent statistically significant more time in their extra cages than mice from the complexity condition, we did not find distinct differences between the complexity and novelty conditions. However, we saw that mice spent more time in the structurally enriched extra cages (complexity and novelty) compared to the structurally non-enriched extra cages (space) and that mice from the complexity enrichment condition reduced their latency to enter the extra cage faster than mice from the structurally non-enriched condition (space).

This is in line with the study by Gross et al. (41), who investigated the effects of different housing conditions on stereotypic and anxiety-related behaviors in mice. They, too, did not find systematic differences between the novelty and complexity condition. In contrast, Abou-Ismail and Mendl (39) found that their complexity housing condition was superior to their novelty housing condition. This discrepancy between the study by Abou-Ismail and Mendl (39) on one hand and the present study on the other could be due to species-specific differences between mice and rats. However, we think that their findings are more likely related to the study’s experimental design. As already mentioned in the introduction, a plausible explanation is their non-permanent provision of nesting material in their novelty housing condition compared to their complexity condition, where nesting material was always available to the rats. The findings of the two studies led us to use a double cage system, in which we could provide constant access to nesting material in the mice’s home cages while offering different levels of enrichment in a voluntarily accessible extra cage rather than the home cage itself.

Our other results indicate a preference for the structurally enriched conditions (complexity and novelty) over the structurally non-enriched space condition, a preference which has been reported previously (35, 68). As it has been argued that preferences can be used to give welfare insights (32–34), a preference for the enriched extra cages over the structurally non-enriched space extra cage might indicate that additional space alone is less beneficial for the mice than increased complexity and novelty. Apart from the differences in preferences, relative body weight was the only other outcome measure affected by enrichment condition. However, we advise caution when interpreting this result. *Post hoc* pairwise comparisons did not reveal any statistically significant differences between the groups. Furthermore, our mice were all non-breeding, healthy, adult females, and unlike young or breeding mice, our mice did not need to gain weight, and their body weight fluctuations were mild and within the normal range.

Apart from a preference for the structurally enriched extra cages (complexity and novelty) over the structurally non-enriched extra cages (space) and the differences in body weight, our results did not reveal further effects of enrichment condition. Albeit not the focus of our study, as the rationale for including different mouse strains was to be better able to generalize our findings, we also tested for strain differences. In the literature, behavioral differences between C57BL/6J and DBA/2N mice have been reported (69). There are also studies showing that the effects of environmental enrichment can be strain-specific (29, 49, 70) and that the presence of the other strain can have an influence as well (45). In our study, however, we did not detect statistically significant differences between the strains. Even so, we cannot conclude that there were no strain differences, as our sensitivity analysis indicated that we could only detect strain effects of large effect sizes (Cohen’s *f* ≥ 0.481).

Overall, the spontaneous behaviors either did not show differences between the groups or the behaviors happened too rarely to be statistically analyzed. Nonetheless, the behavioral data we gathered gave some insights. For instance, the occurrence of bar mouthing, stereotypic and agonistic behavior was very low, and we saw play behavior in all three housing conditions despite the mice’s relatively advanced age (PND 167–202), albeit rarely. Play behavior has been reported to be a reliable indicator of good welfare, whereas weight loss, inactivity, and the presence of stereotypic and agonistic behavior are considered signs of impaired welfare (32, 46, 47). Together with the mice’s relatively stable weight, our observations indicated that the furnishing we offered in the home cages was already of a relatively high standard and provided the mice with good welfare, even though definite claims cannot be made here, as in addition to the provided enrichment in the home cages, all mice had regular access to at least extra space. As the authors of previous studies already pointed out, the provision of nesting material is already sufficient to improve the welfare of laboratory mice greatly (10, 41). Likewise, it has been argued that providing enrichment diversity, i.e., complexity, is probably more important for the animals’ welfare than providing novelty without diversity (39).

In conclusion, our study showed that additional structural enrichment was more attractive to mice than extra space alone. However, with only one result indicating that novelty was preferred over complexity, the present study does not allow us to determine whether or not enrichment novelty increased mouse welfare beyond the effects of enrichment complexity. We did not detect differences between the two enrichment conditions in mice, but it is still conceivable that there are effects of novelty beyond complexity, especially regarding extended periods and with a wider range of variables assessed. To answer this question is of great interest not only for mice and other lab animals. Rather, this concern regards all captive animals, be it in zoos, on farms, or in our own homes. Further research is needed to explore whether additional novelty may be more refining than environmental complexity alone.

## Data availability statement

The raw data supporting the conclusions of this article will be made available by the authors, without undue reservation.

## Ethics statement

The animal study was approved by the local (Amt für Gesundheit, Veterinär- und Lebensmittelangelegenheiten, Münster, Nordrhein-Westfalen, reference number: 39.32.7.1) and federal authorities (Landesamt für Natur, Umwelt und Verbraucherschutz Nordrhein-Westfalen “LANUV NRW”). The study was conducted in accordance with the local legislation and institutional requirements.

## Author contributions

SK, SR, and NS conceived the study. LBi, NK, SK, SR, and NS designed the experiments. LBi carried out the experimental procedures. LBo and VK analyzed the data. LBo prepared the manuscript. All authors reviewed and approved the final version of the manuscript.
