# Supplementary material for: Structural enrichment for laboratory mice: exploring the effects of novelty and complexity
Source: Front Vet Sci. 2023 Sep 29;10:1207332. doi: 10.3389/fvets.2023.1207332 (PMC10570735; doi:10.3389/fvets.2023.1207332)
Supplement: Supplementary file 1 [file Data_Sheet_1.pdf]

Supplementary Material for

“Structural enrichment for laboratory mice: exploring the effects of novelty and complexity”

Lena Bohn[1,2,*], Louisa Bierbaum[1,2], Niklas Kästner[1,2], Vanessa Tabea von Kortzfleisch[1], Sylvia Kaiser[1,2], Norbert Sachser[1,2], S. Helene Richter[1,2,*]

[1]Department of Behavioural Biology, Institute of Neuro- and Behavioural Biology, University of Münster, Münster, Germany.

[2]Münster Graduate School of Evolution, University of Münster, Germany.

*** Correspondence:**
Lena Bohn, lena.bohn@uni-muenster.de
S. Helene Richter, richterh@uni-muenster.de

CONTENTS

Supplementary Figure 1: Percentage of observations with inactivity, and mean of individual counts of extra cage enrichment item interaction, lid climbing frequency, digging frequency, and playing frequency

Supplementary Figure 2: Descriptively analysed behaviours

Supplementary Table 1: Statistical analysis of enrichment condition effect on spontaneous behaviours

Supplementary Table 2: Statistical analysis of latency to enter the extra cage and relative body weight

Supplementary Table 3: Post hoc analysis for latency to enter the extra cage and relative body weight

Supplementary Table 4: Statistical analysis of strain effect on behaviours.

Supplementary Table 5: Statistical analysis of week effect on behaviours.

Supplementary Table 6: Post hoc analysis of week effect on behaviours.

Supplementary Table 7: Summary statistics of descriptively analysed spontaneous behaviours.

Supplementary Table 8: Sensitivity analysis

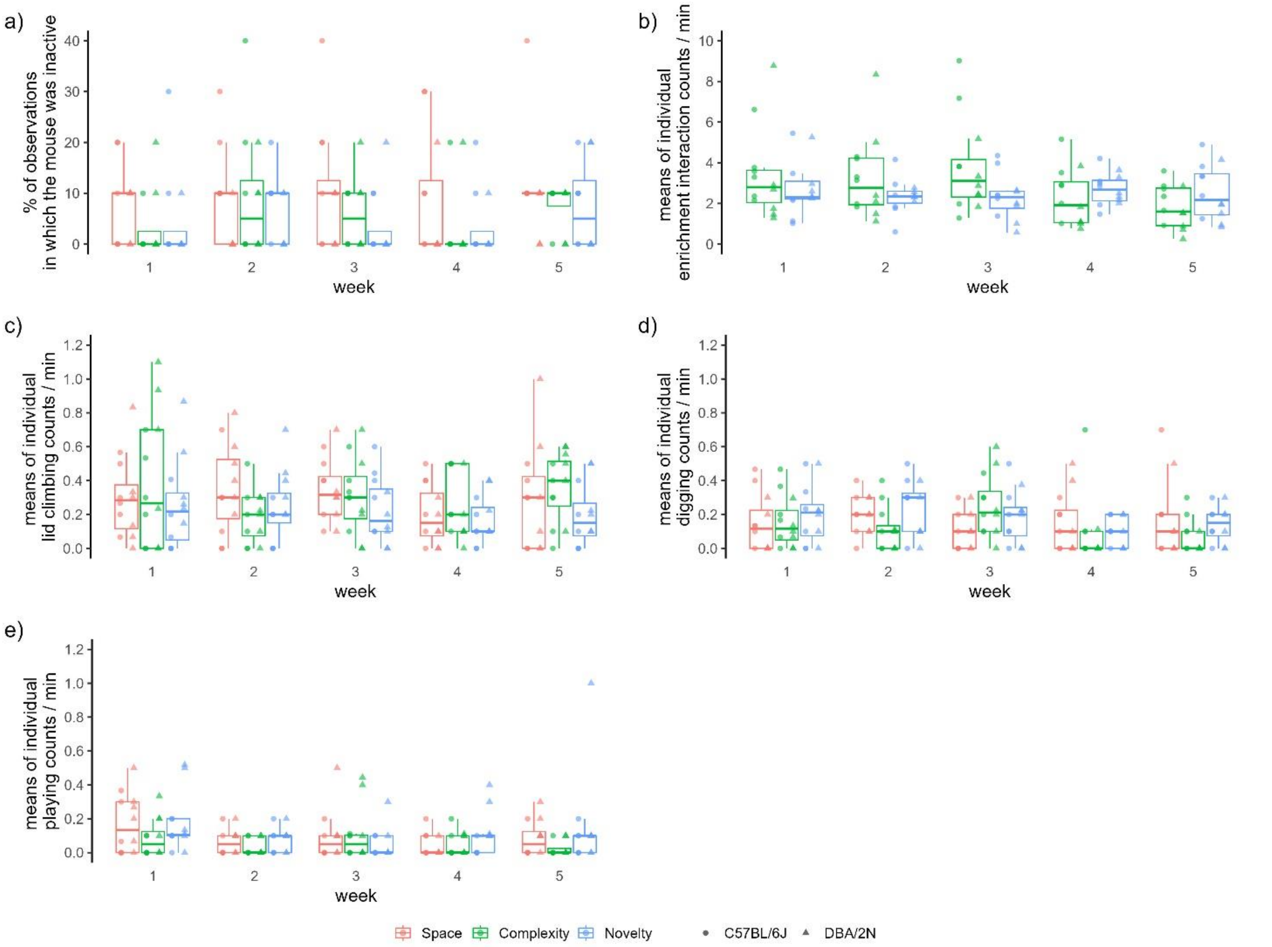

Supplementary Figure 1: Percentage of observations with inactivity, mean of individual counts of extra cage enrichment item interaction, lid climbing frequency, digging frequency, and playing frequency. The boxplots show the median, upper and lower quartile of the respective group. The colours refer to enrichment condition (red = space, green = complexity, blue = novelty), and dot shapes refer to strains (round = C57BL/6J, triangular = DBA/2N). We used $N_{C57BL/6J,\ space} = N_{C57BL/6J,\ complexity} = N_{C57BL/6J,\ novelty} = N_{DBA/2N,\ space} = N_{DBA/2N,\ complexity} = N_{DBA/2N,\ novelty} = 6$ individuals per group, all non-breeding females. Each dot represents the average of one individual each week, with ten measurements per individual and week. The increments in (a) are caused by inactivity being assessed using 1/0 sampling, in contrast to the other spontaneous behaviours which are assessed by counting. The space enrichment group is not included in (b), as the extra cages did not provide enrichment items to interact with.

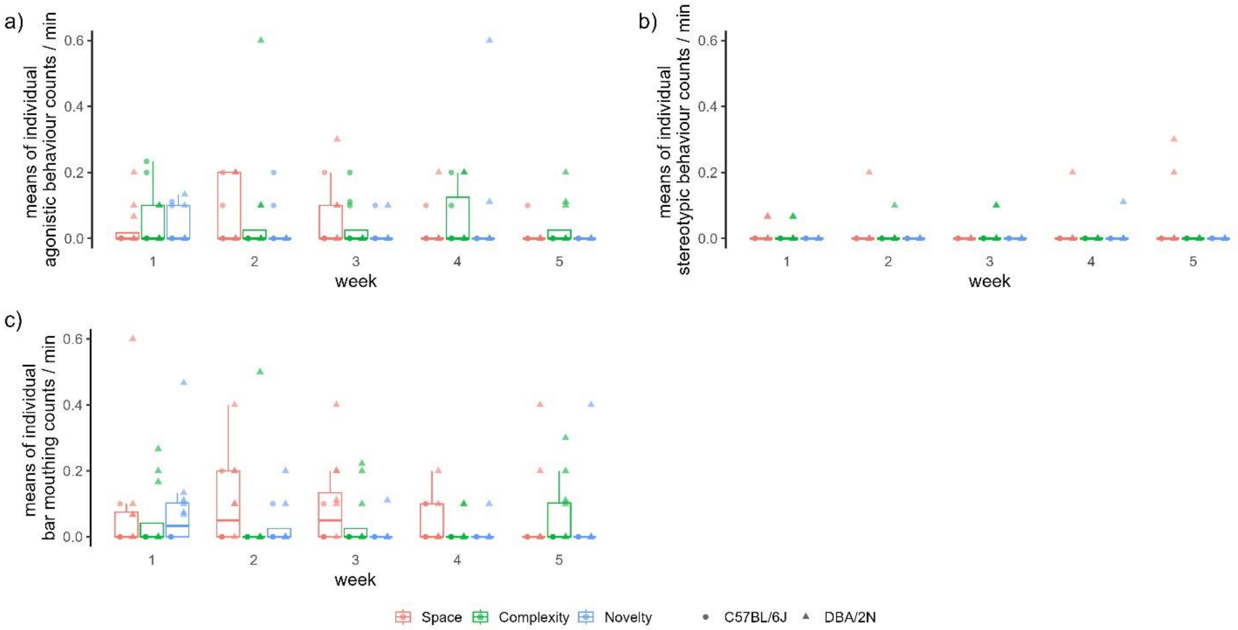

Supplementary Figure 2: Descriptively analysed behaviours. The boxplots show the median, upper and lower quartile of the respective group. The colours refer to enrichment condition (red = space, green = complexity, blue = novelty), and dot shapes refer to strains (round = C57BL/6J, triangular = DBA/2N). We used $N_{C57BL/6J,\ space} = N_{C57BL/6J,\ complexity} = N_{C57BL/6J,\ novelty} = N_{DBA/2N,\ space} = N_{DBA/2N,\ complexity} = N_{DBA/2N,\ novelty} = 6$ individuals per group, all non-breeding females. Each dot represents the average of one individual each week, with ten measurements per individual and week.

Supplementary Table 1: Statistical analysis of enrichment condition effect on spontaneous behaviours. To test for an effect of enrichment condition (space, complexity, and novelty) in each week separately and across all weeks together, we applied Kruskal-Wallis rank sum tests using the kruskal.test function in R. Because the extra cage of the space housing condition did not offer any enrichment items, we applied Mann-Whitney-U tests using the wilcox.test function in R to analyse extra cage enrichment item interaction frequency. In case of a significant Kruskal-Wallis test result, we performed post hoc analysis using the dunn.test function in R for the pairwise comparison. Differences were considered statistically significant at $p < 0.05$ (**bold**). We used sequential Bonferroni-Holm correction (Holm, 1979) to adjust the p-values after post hoc pairwise comparison, as well as after Kruskal-Wallis tests to account for multiple hypothesis testing when comparing enrichment conditions in individual weeks. Shown are the test statistics χ^2 from the Kruskal-Wallis tests (df=2) or W from the Mann-Whitney-U tests, respectively, and Z values from the Dunn tests. Numbers of individuals per group were $N_{C57BL/6J,\ space} = N_{C57BL/6J,\ complexity} = N_{C57BL/6J,\ novelty} = N_{DBA/2N,\ space} = N_{DBA/2N,\ complexity} = N_{DBA/2N,\ novelty} = 6$, with ten measurements per individual and week. Values were averaged for individual and week so that we had one value (the mean) for each individual and week.

**Main effect of enrichment condition**				**Post hoc pairwise comparison**					
				Complexity – Novelty		Complexity – Space		Novelty – Space	
	**χ^2 or W**	**df**	**p value**	**Z**	**p value**	**Z**	**p value**	**Z**	**p value**
**Percentage of time spent in the extra cage**									
Week 1	12.578	2	**0.008**	0.794	0.213	3.391	**0.001**	2.596	**0.009**
Week 2	11.084	2	**0.012**	-0.901	0.184	2.325	**0.020**	3.226	**0.002**
Week 3	14.114	2	**0.005**	-2.558	**0.011**	1.104	0.135	3.662	**<0.001**
Week 4	2.958	2	0.228	-	-	-	-	-	-
Week 5	8.997	2	**0.022**	-0.271	0.393	2.451	**0.014**	2.723	**0.010**
Weeks 1-5	16.294	2	**<0.001**	-1.472	0.070	2.519	**0.012**	3.991	**<0.001**
**Extra cage enrichment item interaction frequency**									
Week 1	84	-	0.514	-	-	-	-	-	-
Week 2	89	-	0.694	-	-	-	-	-	-
Week 3	101	-	0.505	-	-	-	-	-	-
Week 4	50	-	0.767	-	-	-	-	-	-
Week 5	50	-	0.767	-	-	-	-	-	-
Weeks 1-5	82	-	0.590	-	-	-	-	-	-
**Percentage of observations with inactivity**									
Week 1	3.295	2	0.960	-	-	-	-	-	-
Week 2	0.059	2	0.971	-	-	-	-	-	-
Week 3	2.997	2	0.892	-	-	-	-	-	-
Week 4	2.048	2	1	-	-	-	-	-	-
Week 5	0.909	2	1	-	-	-	-	-	-
Weeks 1-5	2.471	2	0.291	-	-	-	-	-	-
**Lid climbing frequency**									
Week 1	0.586	2	0.746	-	-	-	-	-	-
Week 2	2.781	2	0.996	-	-	-	-	-	-
Week 3	2.046	2	1	-	-	-	-	-	-
Week 4	1.246	2	1	-	-	-	-	-	-
Week 5	3.632	2	0.815	-	-	-	-	-	-
Weeks 1-5	2.186	2	0.335	-	-	-	-	-	-

Digging frequency										
Week 1	1.046	2	0.593	-	-	-	-	-	-
Week 2	4.418	2	0.440	-	-	-	-	-	-
Week 3	4.624	2	0.495	-	-	-	-	-	-
Week 4	2.627	2	0.538	-	-	-	-	-	-
Week 5	3.026	2	0.660	-	-	-	-	-	-
Weeks 1-5	2.713	2	0.258	-	-	-	-	-	-
**Playing frequency**									
Week 1	2.494	2	1	-	-	-	-	-	-
Week 2	2.139	2	1	-	-	-	-	-	-
Week 3	1.152	2	0.562	-	-	-	-	-	-
Week 4	1.914	2	0.768	-	-	-	-	-	-
Week 5	3.491	2	0.875	-	-	-	-	-	-
Weeks 1-5	1.581	2	0.454	-	-	-	-	-	-

Supplementary Table 2: Statistical analysis of latency to enter the extra cage and relative body weight. Analysis was based on linear mixed-effects models, using the lmer function in R. The models included three fixed factors (enrichment condition, strain, and week) and their interactions, namely enrichment condition*strain and enrichment condition*week, with week as a numeric factor. We used the C57BL/6J strain and the space enrichment condition as model reference level. Mouse ID was included as a random factor. To calculate F-statistic and p-values for the fixed factors and interactions, ANOVA type III tables were produced with the Satterthwaite method for denominator degrees of freedom using the anova function in R. Differences were considered statistically significant at $p < 0.05$ (**bold**). Transformation of the response variable (y) was applied for latency to enter the extra cage, using Tukey's Ladder of Power with the transformTukey function in R. Numbers of individuals per group were $N_{C57BL/6J,\ space} = N_{C57BL/6J,\ complexity} = N_{C57BL/6J,\ novelty} = N_{DBA/2N,\ space} = N_{DBA/2N,\ complexity} = N_{DBA/2N,\ novelty} = 6$, with ten measurements per individual and week for latency to enter the home cage, and 1 measurement per individual and week for relative body weight.

Fixed factors	Sum Sq	Mean Sq	NumDF	DenDF	F value	p value	Transformation of the response variable (y)
**Latency to enter the extra cage**							
enrichment condition	0.015	0.008	2	101.414	1.025	0.362	$-1*y^{\lambda}$, $\lambda = -0.125$
strain	0.015	0.015	1	30.006	1.980	0.170	
week	7.494	7.494	1	1429.553	1004.212	**<0.001**	
enrichment condition:strain	0.006	0.003	2	30.005	0.425	0.658	
enrichment condition:week	0.072	0.036	2	1429.553	4.828	**0.008**	
**Relative body weight**							
enrichment condition	48.780	24.387	2	134.10	3.37	**0.037**	none
strain	19.023	19.023	1	30	2.63	0.116	
week	5.569	5.569	1	105	0.77	0.383	
enrichment condition:strain	25.927	12.963	2	30	1.79	0.184	
enrichment condition:week	58.460	29.230	2	105	4.04	**0.020**	

Supplementary Table 3: Post hoc analysis for latency to enter the extra cage and relative body weight. We conducted pairwise comparisons for the statistically significant results from the ANOVA, namely the effect of enrichment condition*week interaction on both latency to enter the extra cage and relative body weight, as well as for the significant main effect of enrichment condition on relative body weight. We used the emtrends and pairs function in R for the effect of enrichment condition*week interaction, or conducted Tukey adjusted pairwise comparisons using the emmeans function in R for the effect of enrichment condition. Shown in the table are the adjusted p values. Differences were considered statistically significant at $p < 0.05$ (**bold**).

Contrasts	Estimates	SE	df	T ratio	p value
**Latency to enter the extra cage: enrichment condition*week interaction**					
Space – Complexity	0.012	0.004	1429.334	2.956	**0.009**
Space – Novelty	0.010	0.004	1429.702	2.345	0.050
Complexity – Novelty	-0.002	0.004	1423.661	-0.597	0.822
**Relative body weight: enrichment condition*week interaction**					
Space – Complexity	-1.107	0.491	105	-2.253	0.067
Space – Novelty	-1.290	0.491	105	-2.625	**0.027**
Complexity – Novelty	-0.183	0.491	105	-0.372	0.927
**Relative body weight: enrichment condition**					
Space – Complexity	0.785	1.01	30	0.778	0.719
Space – Novelty	-0.236	1.01	30	-0.234	0.970
Complexity – Novelty	-1.022	1.01	30	-1.012	0.575

Supplementary Table 4: Statistical analysis of strain effect on behaviours. To test for an effect of strain (C57BL/6J and DBA/2N) on our behavioural parameters in each enrichment condition, we applied Mann-Whitney-U tests using the wilcox.test function in R. To account for multiple hypothesis testing when comparing the two strains in each enrichment condition, we used sequential Bonferroni-Holm correction (Holm, 1979) to adjust the p values. P values shown in the table are the adjusted p values. Differences were considered statistically significant at $p < 0.05$ (**bold**). Numbers of individuals per group were $N_{C57BL/6J,\ space} = N_{C57BL/6J,\ complexity} = N_{C57BL/6J,\ novelty} = N_{DBA/2N,\ space} = N_{DBA/2N,\ complexity} = N_{DBA/2N,\ novelty} = 6$, with ten measurements per individual and week. Values were averaged for individual and weeks.

	W	p value
**Percentage of time spent in the extra cage**		
Space	23	0.485
Complexity	24	0.985
Novelty	12	0.985
**Extra cage enrichment item interaction frequency**		
Space	-	-
Complexity	27	0.360
Novelty	25	0.310
**Percentage of observations with inactivity**		
Space	32	0.087
Complexity	19	0.934
Novelty	27.5	0.280
**Lid climbing frequency**		
Space	17	0.937
Complexity	14	1
Novelty	9	0.540
**Digging frequency**		
Space	19.5	0.872
Complexity	29	0.276
Novelty	24.5	0.668
**Playing frequency**		
Space	8	0.126
Complexity	7.5	0.321
Novelty	8	0.254

Supplementary Table 5: Statistical analysis of week effect on behaviours. To test for an effect of week on our behavioural parameters in each enrichment condition, we applied Friedman rank sum tests using the friedman.test function in R. To account for multiple hypothesis testing when comparing the five weeks in each condition, we used sequential Bonferroni-Holm correction (Holm, 1979) to adjust the p values. P values shown in the table are the adjusted p values. Differences were considered statistically significant at $p < 0.05$ (**bold**). Numbers of individuals per group were $N_{C57BL/6J,\ space} = N_{C57BL/6J,\ complexity} = N_{C57BL/6J,\ novelty} = N_{DBA/2N,\ space} = N_{DBA/2N,\ complexity} = N_{DBA/2N,\ novelty} = 6$, with ten measurements per individual and week. Values were averaged for individual and week.

	W	df	p value
**Percentage of time spent in the extra cage**			
Space	0.333	4	0.988
Complexity	5.333	4	0.765
Novelty	4	4	0.812
**Extra cage enrichment item interaction frequency**			
Space	-		-
Complexity	12.333	4	**0.030**
Novelty	3.067	4	0.547
**Percentage of observations with inactivity**			
Space	3.167	4	0.530
Complexity	5.258	4	0.696
Novelty	4.652	4	0.650
**Lid climbing frequency**			
Space	6.044	4	0.588
Complexity	3.947	4	0.413
Novelty	5.059	4	0.562
**Digging frequency**			
Space	5.703	4	0.222
Complexity	16.485	4	**0.006**
Novelty	5.909	4	0.412
**Playing frequency**			
Space	4.462	4	0.347
Complexity	4.949	4	0.879
Novelty	8.418	4	0.154

Supplementary Table 6: Post hoc analysis of week effect on behaviours. For the statistically significant results from the Friedman test, namely the effect of week on the extra cage enrichment item interaction frequency in the complexity enrichment condition and the effect of week on the digging frequency in the complexity enrichment condition, we conducted Bonferroni-Holm adjusted pairwise comparisons using the paired wilcox.test in R. P values shown in the table are the adjusted p values. Differences were considered statistically significant at $p < 0.05$ (**bold**).

Comparison	V	p value
**Extra cage enrichment item interaction frequency: Complexity enrichment condition**		
Week 1 - Week 2	42	0.850
Week 1 - Week 3	32	1
Week 1 - Week 4	55	0.932
Week 1 - Week 5	66	0.272
Week 2 - Week 3	26	1
Week 2 - Week 4	55	1
Week 2 - Week 5	64	0.364
Week 3 - Week 4	68	0.189
Week 3 - Week 5	72	0.070
Week 4 - Week 5	63	0.384
**Digging frequency: Complexity enrichment condition**		
Week 1 - Week 2	28	1
Week 1 - Week 3	12	0.882
Week 1 - Week 4	41.5	0.845
Week 1 - Week 5	41.5	1
Week 2 - Week 3	7.5	0.414
Week 2 - Week 4	20	1
Week 2 - Week 5	32.5	1
Week 3 - Week 4	55	0.440
Week 3 - Week 5	52	0.130
Week 4 - Week 5	12	0.832

Supplementary Table 7: Summary statistics of descriptively analysed spontaneous behaviours. Some behaviours, namely agonistic behaviour, stereotypic behaviour, and bar mouthing happened too rarely to be statistically analysed. Instead, for those behaviours we show summary statistics. Numbers of individuals per group were $N_{C57BL/6J, space} = N_{C57BL/6J, complexity} = N_{C57BL/6J, novelty} = N_{DBA/2N, space} = N_{DBA/2N, complexity} = N_{DBA/2N, novelty} = 6$, with then measurements per individual and week. Values were averaged for individual, week, and enrichment condition.

	median	mean	range (min – max)	standard deviation	variance
**Agonistic behaviour frequency**					
Week 1	0	0.040	0 – 0.233	0.068	0.005
Week 2	0	0.056	0 – 0.6	0.118	0.014
Week 3	0	0.036	0 – 0.3	0.073	0.005
Week 4	0	0.048	0 – 0.6	0.116	0.013
Week 5	0	0.014	0 – 0.2	0.043	0.002
**Stereotypic behaviour frequency**					
Week 1	0	0.007	0 – 0.067	0.021	0
Week 2	0	0.008	0 – 0.2	0.037	0.001
Week 3	0	0.006	0 – 0.1	0.023	0.001
Week 4	0	0.009	0 – 0.2	0.038	0.001
Week 5	0	0.014	0 – 0.3	0.059	0.004
**Bar mouthing frequency**					
Week 1	0	0.070	0 – 0.6	0.133	0.018
Week 2	0	0.058	0 – 0.5	0.118	0.014
Week 3	0	0.048	0 – 0.4	0.093	0.009
Week 4	0	0.022	0 – 0.2	0.048	0.002
Week 5	0	0.048	0 – 0.4	0.111	0.012

Supplementary Table 8: Sensitivity analysis. Using the software G*Power, we approximated the detectable effect sizes f for linear mixed effect models with interactions (LMMs). This analysis shows that with the data and analysis at hand, we should be able to detect effect sizes equal to or larger than indicated in the table with a power of 80 % and an α error probability of 5%. The total sample size was N = 36.

Fixed factors	detectable effect sizes f
enrichment condition	0.542
strain	0.481
week	0.185
enrichment condition:strain	0.544
enrichment condition:week	0.210
